# Non‐verbal dichotic listening: A new cognitive hearing test for dementia

**DOI:** 10.1002/alz.71358

**Published:** 2026-05-26

**Authors:** Chris J. D. Hardy, Benjamin A. Levett, Jessica Jiang, Lucy B. Core, Sophie Froud, Lauren Bishop, Dicky Lim, Faiza Durrani, Anna Volkmer, Nehzat Koohi, Doris‐Eva Bamiou, Charles R. Marshall, Jason D. Warren

**Affiliations:** ^1^ Dementia Research Centre UCL Queen Square Institute of Neurology, UCL London UK; ^2^ Centre for Preventive Neurology Queen Mary University of London London UK; ^3^ Psychology and Language Sciences (PALS), UCL London UK; ^4^ Department of Clinical and Movement Neurosciences Institute of Neurology, UCL London UK; ^5^ The Ear Institute, UCL London UK

**Keywords:** Alzheimer's disease, central hearing, dementia, dichotic listening, frontotemporal dementia auditory cognition, hearing, primary progressive aphasia

## Abstract

**INTRODUCTION:**

Central hearing difficulties are a feature of Alzheimer's disease (AD) but not well captured by standard speech perception tests.

**METHODS:**

We developed a non‐verbal dichotic listening test (NVDLT) based on everyday sounds, and compared this with a standard verbal dichotic listening test (VDLT) in 36 people with primary progressive aphasia (PPA), 18 with typical AD (tAD), 6 with right temporal‐variant frontotemporal dementia (rtvFTD), and 29 cognitively‐healthy controls. Daily‐life hearing function was assessed using the modified Amsterdam Inventory for Auditory Disability and Handicap (mAIAD).

**RESULTS:**

On NVDLT, all dementia groups except rtvFTD performed worse than controls; tAD, logopenic‐variant PPA and nonfluent/agrammatic‐variant PPA performed worse than rtvFTD. NVDLT performance predicted atrophy in the retrosplenial cortex and hippocampus. NVDLT score discriminated tAD patients from controls with near‐perfect accuracy (area under the curve [AUC] = 0.99) and predicted daily‐life hearing function (*r* = 0.57).

**DISCUSSION:**

NVDLT performance indexes daily‐life hearing function and may aid dementia diagnosis, potentially in culturally‐diverse populations.

## Highlights

- We created a central hearing test for dementia using non‐verbal dichotic listening
- Non‐verbal dichotic listening is differentially affected in major dementias
- The new test detected dementia with excellent accuracy
- It predicted gray matter loss in core brain circuitry targeted by Alzheimer's disease
- Non‐verbal dichotic listening holds promise as a culture‐fair marker of dementia

## 1 BACKGROUND

The association between hearing impairment and dementia may in part reflect auditory brain dysfunction due to neurodegenerative pathologies.^1^ Patients with typical Alzheimer's disease (tAD) and its variants have difficulties with auditory scene analysis (ASA),^2^, ^3^ reflecting involvement of the temporo‐parietal default‐mode network targeted by AD pathology.^4^ Dichotic listening—disambiguating two different sounds presented simultaneously to each ear—is commonly used to assess ASA. Speech‐based dichotic tests show deficits in early tAD and other dementias.^1^, ^5^, ^6^, ^7^, ^8^, ^9^, ^10^ Non‐verbal analogues are feasible and potentially culture‐fair^11^, ^12^ but have not been explored in dementia.

Here we created a non‐verbal dichotic listening test (NVDLT) and assessed its performance in relation to a verbal dichotic listening test (VDLT)^13^ in patients with dementia syndromes (tAD, canonical syndromes of primary progressive aphasia [PPA], and right temporal variant frontotemporal dementia [rtvFTD]) compared with healthy older participants. VDLT deficits have been documented in tAD and PPA, most markedly in the nonfluent/agrammatic variant (nfvPPA) and logopenic variant (lvPPA),^6^, ^8^, ^10^ reflecting the targeting of temporo‐parietal ASA mechanisms in these syndromes. We hypothesised that NVDLT (like VDLT) performance would be more severely affected in tAD, nfvPPA, and lvPPA than in semantic variant primary progressive aphasia (svPPA) and rtvFTD, and would predict daily‐life hearing function. Using voxel‐based morphometry (VBM), we assessed whether NVDLT and VDLT performance predicted regional gray matter atrophy in default mode and auditory networks. ^3^, ^8^, ^14^, ^15^, ^16^, ^17^, ^18^, ^19^, ^20^, ^21^, ^22^

## 2 Methods

### 2.1 Participants

Eighteen patients with tAD, 14 with lvPPA, 10 with nfvPPA, 12 with svPPA, and 6 with rtvFTD were recruited via a specialist cognitive clinic. All patients fulfilled criteria^23^, ^24^, ^25^ for the relevant syndrome with clinically mild‐to‐moderate disease, supported by brain MRI. Twenty‐nine cognitively‐well controls with no history of neurological/psychiatric disorders also participated.

Each participant had pure‐tone audiometry (from which we derived a four‐frequency pure‐tone audiometry better ear average threshold (PTA‐BEA)), and a neuropsychological assessment (Supplementary Materials).

All participants gave written informed consent. Ethical approval was granted by the University College London‐National Hospital for Neurology and Neurosurgery (UCL‐NHNN) Joint Research Ethics Committee, following Declaration of Helsinki guidelines.

### 2.2 Hearing assessments

The VDLT was a standard dichotic digits test.^13^ On each trial, two pairs of different digits were presented, one to the left and the other simultaneously to the right ear; the task was to repeat the four digits heard, in any order (Supplementary Materials).

The NVDLT was based on widely familiar non‐verbal sounds, without strong linguistic and/or cultural associations (Figures S1 and S2) based on pilot work (Table S1). On each trial, two pairs of different sounds were presented, one to the left and the other simultaneously to the right ear; the task was to identify the four sounds heard, in any order, using a response picture matrix (Figure S2). Participants were not allowed to point to pictures on the matrix until all sounds had finished playing.

Daily‐life hearing function was assessed using the modified Amsterdam Inventory for Auditory Disability and Handicap (mAIAD)^26^ (Supplementary Materials).

### 2.3 Analysis of clinical and neuropsychological data

Data were analyzed in Stata v14. For continuous demographic and neuropsychological data, participant groups were compared using analyses of variance (ANOVA); categorical data were compared using Fisher's exact tests. Where the omnibus test showed a significant effect of diagnosis, pairwise comparisons were conducted to identify the groups driving the effect.

### 2.4 Analysis of behavioral data

We analyzed performance on the VDLT and NVDLT using analysis of covariance (ANCOVA) models adjusting for covariates of age, sex, PTA‐BEA, and Mini‐Mental State Examination (MMSE) score. We also compared VDLT and NVDLT directly by converting each to a percentage score and subtracting NVDLT from VDLT, using this as dependent variable in the same model. We ran the same models removing participants with digit span ≤4, and excluding participants with asymmetrical hearing loss (see Supplementary Materials).

We tested for a right ear advantage (REA) for verbal material and left ear advantage (LEA) for non‐verbal material^27^, ^28^ (Supplementary Materials).

Correlations between VDLT and NVDLT scores and each with PTA‐BEA and forward digit span were assessed using Pearson's correlations, in the control and combined patient groups separately. Correlations of VDLT, NVDLT, MMSE score, and PTA‐BEA with mAIAD scores were assessed using Pearson's correlations across all participants combined.

Receiver‐operating characteristic (ROC) curves were derived to assess overall diagnostic utility of the VDLT and NVDLT in distinguishing all patients from controls, and patients with typical AD from controls. We compared areas under the curve (AUCs) generated for the VDLT and NVDLT with PTA‐BEA and MMSE score.

### 2.5 Brain image acquisition and analysis

Volumetric brain MRI scans were acquired for 55 patients (18 AD, 12 lvPPA, 8 nfvPPA, 11 svPPA, 6 rtvFTD) and entered into a VBM analysis. Regional gray matter associations of VDLT, NVDLT, and differential test performance were assessed across the patient cohort in a regression model, adjusting for nuisance covariates of diagnosis, age, sex, total intracranial volume, MMSE score, and PTA‐BEA, at significance threshold p < 0.05 after family‐wise error (FWE) correction for multiple voxel‐wise comparisons within regions of interest (ROIs) based on prior neuroanatomic hypotheses (Figure S3; further details in Supplementary Materials).

**RESEARCH IN CONTEXT**

1. **Systematic review**: We reviewed the literature using traditional sources, meeting abstracts and presentations. Although verbal dichotic listening tests (VDLTs) have been studied previously in people with dementia, here we created a new, non‐verbal dichotic listening test (NVDLT) for dementia based on everyday sounds that promises to be more inclusive and culture‐fair.
2. **Interpretation**: Non‐verbal dichotic listening is a novel, sensitive marker of AD that predicts daily‐life hearing function, potentially applicable across culturally‐ and linguistically‐diverse populations.
3. **Future directions**: Future research should assess the impact of different sounds and sound combinations (in particular, sounds with frequencies within the range of human speech) on non‐verbal dichotic listening test difficulty, and the potentially confounding effects of presbycusis. It will also be essential to directly evaluate the culture‐fairness of the NVDLT in larger cohorts representing linguistically and culturally diverse older populations.

## 3 Results

**FIGURE 1:**
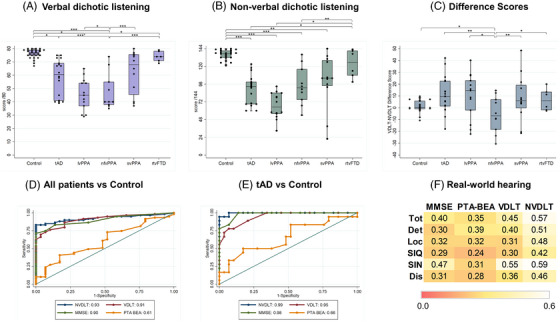
(A–**C)** Box‐and‐whisker plots showing verbal dichotic listening (**A**), non‐verbal dichotic listening (**B**), and verbal minus non‐verbal dichotic listening difference (**C**) scores for all participant groups. Raw scores are presented for the first two panels; the difference scores panel shows percentage difference scores after converting raw scores on each of the dichotic listening tests to percentages and subtracting non‐verbal dichotic listening performance from dichotic listening performance. Boxes code interquartile range; whiskers code 95% confidence intervals; transverse lines code median scores; dots show individual scores. Significant between‐group differences (after adjustment for covariates) are represented using horizontal brackets above the relevant groups; *p < 0.05; **p < 0.01; ***p < 0.001. (**D,E**) ROC curves for combined patient groups versus healthy controls (**D**) and patients with typical Alzheimer's disease (tAD) versus healthy controls (**E**), based on binary classifiers of NVDLT score, VDLT score, MMSE score, and PTA‐BEA. AUCs are shown for each classifier below each panel; an AUC of 1 would correspond to an ideal classifier. (**F)** Heat maps showing correlations between subdomain scores (*y*‐axis) on the mAIAD questionnaire and auditory test performance (*x*‐axis) across the combined cohort. Each cell shows the Pearson correlation coefficient between the score on the relevant questionnaire subscale and the auditory test score; paler colors code increasing strength of correlation, as indexed on the color bar. Dis, Discrimination = items exploring auditory discrimination (e.g., do you recognize your family members by their voices?); Loc, localization = items exploring auditory spatial localization (e.g., do you hear from what direction a car horn is coming?); SIN, speech‐in‐noise = items exploring speech understanding in noisy environments (e.g., can you understand a shop assistant in a crowded shop?); SIQ, speech‐in‐quiet = items exploring speech understanding in quiet (e.g., can you understand the presenter of the news on TV?); Det, detection = items exploring sound detection (e.g., can you hear the doorbell at home?); Tot, total = correlation coefficient across all items on mAIAD. Control, healthy older volunteer group; lvPPA, patient group with logopenic variant primary progressive aphasia (PPA); MMSE, Mini‐Mental State Examination; nfvPPA, patient group with nonfluent/agrammatic variant PPA; NVDLT, non‐verbal dichotic listening test; rtvFTD, patient group with right temporal variant frontotemporal dementia; svPPA, patient group with semantic variant PPA; tAD, patient group with typical Alzheimer's disease; VDLT, verbal dichotic listening test.

General participant and neuropsychological performance data by group are presented in Table 1; dichotic listening characteristics are presented in Table 2 and Figure 1. Further details of hearing test comparisons are in Table S2.

**TABLE 1:** General demographic, clinical, and neuropsychological characteristics of participant groups.

Characteristic	Control (*n* = 29)	AD (*n* = 18)	lvPPA (*n* = 14)	nfvPPA (*n* = 10)	svPPA (*n* = 12)	rtvFTD (*n* = 6)	Omnibus test
**Demographic and clinical**							
Age, *y*	70.0 (6.6)	73.3 (6.9)	69.1 (6.9)	74.2 (7.4)	64.4(9.4)^‡^, ^§^	69.2 (4.1)	*χ* ^2^(5) = 12.3, *p* = 0.03
Sex (F:M)	21:8	9:9	**2:12**	5:5	**4:8**	**1:5**	*p* = 0.005
Education, y	15.7 (2.5)	15.2 (3.0)	14.8 (1.9)	13.9 (1.5)	16.3 (2.1)	15.5 (3.2)	*χ* ^2^(5) = 8.0, *p* = 0.16
Hand (L:R:A)	2:26:1	1:17:0	3:11:0	1:9:0	4:8:0	3:3:0	*p* = 0.303
Duration, y	N/A	5.2 (3.0)	5.6 (3.4)	3.8 (4.6)	4.4 (1.9)	5.2 (3.3)	*χ* ^2^(4) = 1.0, *p* = 0.91
MMSE (/30)	28.8 (1.3)	**21.2 (4.8)** ^*^	**13.7 (4.6)** ^*^ **^,^** ^†^ **^,^** ^‡^ **^,^** ^§^	**25.4 (3.0)**	**21.3 (8.5)**	27.3 (3.1)	*χ* ^2^(5) = 57.6, *p* < 0.001
PTA BEA, dB	18.4 (9.0)	25.9 (14.4)	21.0 (14.7)	26.1 (8.1)	19.7 (8.7)	24.0 (19.4)	*χ* ^2^(5) = 7.9, *p* = 0.2
**Modified Amsterdam Inventory of Auditory Disability and Handicap**							
Total (/112)	105.3 (5.4)^a^	**94.6 (13.9)**	**87.2 (15.1)** ^a^	**90.1 (15.3)** ^a^	**83.3 (8.6)** ^‡^, ^c^	**89.0 (19.8)**	*χ* ^2^(5) = 31.6, *p* < 0.001
Detection (/20)	19.3 (1.2)^a^	**17.4 (2.5)**	**17.1 (2.6)** ^a^	**17.2 (2.9)** ^a^	**17.0 (2.4)** ^c^	17.7 (2.7)	*χ* ^2^(5) = 16.3, *p* = 0.006
Local (/20)	18.9 (1.5)^a^	17.4 (3.4)	**15.3 (3.6)** ^a^	**16.0 (3.2)** ^a^	16.3 (3.8)^c^	**15.7 (4.13)**	*χ* ^2^(5) = 15.1, *p* = 0.01
SIQ (/20)	18.6 (1.1)^a^	17.1 (2.7)	**16.2 (2.9)** ^a^	17.0 (3.4)^a^	**13.2 (2.1)** ^‡^, ^§^, ^¶^, ^c^	15.5 (4.5)	*χ* ^2^(5) = 23.1, *p* < 0.001
SIN (/20)	18.0 (1.9)^a^	**14.8 (4.0)**	**11.3 (3.1)** ^‡^, ^a^	**12.3 (3.5)** ^a^	**10.2 (2.6)** ^‡^, ^c^	**13.8 (5.1)**	*χ* ^2^(5) = 38.1, *p* < 0.001
Discrim (/32)	30.6 (1.5)^a^	**28.0 (4.2)**	**27.3 (4.3)** ^a^	28.1 (4.8)^a^	**26.6 (2.1)** ^c^	**26.3 (4.8)**	*χ* ^2^(5) = 19.0, *p* = 0.002
**Episodic memory**							
CPAL (/24)	20.8 (2.3)^a^	**3.9 (4.3)** ^*^ **^,^** ^§^, ^b^	**1.6 (2.0)** ^*^ **^,^** ^§^, ^c^	15.6 (7.1)^d^	**4.5 (5.6)** ^*^ **^,^** ^§^, ^d^	19.0 (2.8)	*χ* ^2^(5) = 44.1, *p* < 0.001
CFMT (/18)	17.8 (0.8)^e^	**12.8 (3.6)** ^e^	NA	NA	**15.4 (2.8)**	**15.8 (2.6)**	*χ* ^2^(3) = 34.1, *p* < 0.001
**Working memory**							
DS forward, max	7.2 (0.9)	**6.3 (1.4)**	**3.8 (1.9)** ^*^ **^,^** ^†^, ^‡^	**4.6 (0.9)** ^*^ **^,^** ^†^, ^‡^, ^d^	**6.3 (0.8)**	7.2 (1.3)	*χ* ^2^(5) = 47.2, *p* < 0.001
DS reverse, max	5.3 (1.4)	**3.9 (1.0)** ^*^	**2.6 (1.3**)^*^ **^,^** ^†^, ^‡^	**3.1 (0.8)** ^*^ **^,^** ^†^, ^d^	4.8 (1.3)	5.5 (1.2)	*χ* ^2^(5) = 40.1, *p* < 0.001
SS forward, max	5.7 (1.0)^d^	**4.7 (1.1)**	**3.5 (0.9)** ^*^ **^,^** ^†^, ^‡^, ^e^	**4.1 (0.9)** ^†^	5.4 (0.8)	5.0 (1.1)	*χ* ^2^(5) = 33.9, *p* < 0.001
SS reverse, max	5.3 (0.9)^d^	**4.0 (1.4)** ^*^ **^,^** ^†^	**2.7 (1.4)** ^*^, ^†^, ^‡^, ^e^	**4.0 (1.1)** ^*^ **^,^** ^†^	5.4 (1.1)	5.3 (1.0)	*χ* ^2^(5) = 37.0, *p* < 0.001
**Executive skills**							
WASI matrices (/32)	26.3 (2.3)	**17.5 (7.4)** ^*^ **^,^** ^†^	**10.4 (6.5)** ^*^ **^,^** ^†^	**15.9 (8.6)** ^*^ **^,^** ^†^	23.4 (8.8)	27.7 (3.3)	*χ* ^2^(5) = 47.7, *p* < 0.001
Letter fluency, total	19.3 (4.4)^f^	**11.8 (7.8)** ^c^	**6.0 (6.1)** ^‡^, ^d^	**4.5 (5.2)** ^*^ **^,^** ^‡^, ^e^	**8.7 (5.2)**	13.2 (4.7)	*χ* ^2^(5) = 30.1. *p* < 0.001
Category fluency, total	24.9 (5.0)^f^	**11.7 (6.1)** ^c^	**5.8 (4.0)** ^*^ **^,^** ^‡^ **^,^** ^§^, ^e^	**11.8 (6.0)**	**8.5 (5.5)** ^*^	**16.8 (6.3)**	F(5,64) = 20.9, *p* < 0.001
**Posterior cortical skills**							
JLO (/30)	27.3 2.9)	**21.6 (8.3)** ^†^, ^b^	**18.0 (6.7)** ^*^ **^,^** ^†^ **^,^** ^§^, ^c^	24.7 (6.2)	25.9 (8.8)	28.0 (2.5)	*χ* ^2^(5) = 25.6, *p* < 0.001
VOSP (/20)	18.6 (1.0)	**16.8 (1.9)** ^d^	**15.4 (3.8)** ^c^	**17.4 (1.6)**	17.4 (3.1)	**16.5 (1.9)**	*χ* ^2^(5) = 19.9, *p* = 0.001
GDA (/24)	14.9 (4.5)^f^	**6.2 (5.5)** ^*^, ^c^	**1.1 (1.7)** ^*^, ^†^, ^‡^, ^§^, ^b^	**6.4 (2.8)** ^*^, ^d^	**8.8 (5.8)** ^*^	15.8 (4.1)	*χ* ^2^(5) = 38.5, *p* < 0.001
**Auditory processing**							
PALPA‐3 (/30)	35.9 (0.3)	**35.0 (1.2)** ^e^	**32.6 (6.8)** ^e^	**35.1 (0.9)** ^d^	**34.9 (1.8)**	35.7 (0.6)^c^	*χ* ^2^(5) = 18.0, *p* = 0.003
**Mini‐Linguistic State Examination**							
Total (/100)	99.2 (1.0)^d^	NA	**74.7 (10.6)** ^b^	**78.0 (14.8)** ^d^	**81.8 (10.9)**	NA	*χ* ^2^(3) = 44.6, *p* < 0.001
Motor (/30)	30.0 (0.0)^d^	NA	29.9 (0.3)^b^	**23.1 (3.7)** ^†^, ^¶^, ^d^	30.0 (0.0)	NA	*χ* ^2^(3) = 53.1, *p* < 0.001
Phonology (/30)	29.5 (0.7)^d^	NA	**24.0 (5.0)** ^†^, ^b^	**24.3 (6.1)** ^†^, ^d^	28.1 (2.9)	NA	*χ* ^2^(3) = 27.6, *p* < 0.001
Semantic (/20)	19.9 (0.3)^d^	NA	**13.6 (3.3)** ^b^	**16.7 (5.6)** ^d^	**10.5 (4.5)** ^§^	NA	*χ* ^2^(3) = 46.3, *p* < 0.001
Syntax (/10)	9.7 (0.6)^d^	NA	**4.7 (3.1)** ^b^	**6.7 (1.5)** ^d^	**7.5 (3.0)**	NA	*χ* ^2^(3) = 31.41 *p* < 0.001
WM (/10)	10.0 (0.2)^d^	NA	**3.1 (4.1)** ^b^	**7.0 (3.1)** ^d^	**5.6 (3.9)**	NA	*χ* ^2^(3) = 35.9, *p* < 0.001
**Word retrieval**							
BNT (/30)	29.3 (1.1)^f^	**21.8 (5.6)** ^e^	**13.9 (9.2)** ^*^, ^‡^, ^d^	**22.1 (7.6)** ^d^	**8.6 (6.9)** ^*^, ^‡^, ^§^	**25.3 (1.4)**	*χ* ^2^(5) = 46.5, *p* < 0.001
**Speech repetition**							
Single word repetition (/45)	44.2 (1.0)	41.7 (3.8)^d^	41.5 (6.9)^d^	**30.3 (17.0)** ^†^, ^‡^, ^¶^	42.1 (4.3)	NA	*χ* ^2^(4) = 19.2, *p* = 0.001
GDSR (/10)	9.2 (0.9)	**7.9 (1.6)** ^c^	**4.9 (1.7)** ^†^, ^‡^	**4.8 (2.7)** ^†^, ^‡^	**7.7 (1.9)**	NA	*χ* ^2^(4) = 44.8, *p* < 0.001

*Notes*: Mean (SD) values and raw scores are presented (maximum value possible in parentheses), unless otherwise indicated. Mean (SD) values are given for variables; counts are given for categorical variables. Maximum scores are indicated in parentheses where appropriate. Significant differences (*p* < 0.05) from healthy control group are indicated in bold. All participants were native English speakers with the exception of one patient with nfvPPA who was fluent in English but whose native language was Danish.

Abbreviations: AD, patient group with typical Alzheimer's disease; BNT, Boston Naming Test; CFMT, Cambridge Face Memory Test; Control, healthy older volunteer group; CPAL, Camden Paired Associates Learning (test); Discrim, discrimination; DS, digit span; GDA, graded difficulty arithmetic; GDSR, graded difficulty sentence repetition; JLO, judgment of line orientation; Local, localization; lvPPA, patient group with logopenic variant primary progressive aphasia (PPA); MMSE, Mini‐Mental State Examination; nfvPPA, patient group with nonfluent/agrammatic variant PPA; PALPA, Psycholinguistic Assessment of Language Processing in Aphasia; PTA BEA, pure‐tone audiometry best ear average; rtvFTD, patient group with right temporal variant PPA; SIQ, speech‐in‐quiet; SIN, speech‐in‐noise; SS, spatial span; svPPA, patient group with semantic variant PPA; VOSP, Visual Object and Space Perception Battery (object decision); WASI, Wechsler Abbreviated Scale of Intelligence; WM, working memory.

* Significantly different (*p* < 0.05) from rtvFTD group.

^†^ Significantly different (*p* < 0.05) from svPPA group.

^‡^ Significantly different (*p* < 0.05) from AD group.

^§^ Significantly different (*p* < 0.05) from nfvPPA group.

^¶^ Significantly different (*p* < 0.05) from lvPPA group. superscript numbers indicate number of participants missing.

^a^ Missing data for 15 participants.

^b^ Missing data for 4 participants.

^c^ Missing data for 3 participants.

^d^ Missing data for 1 participant.

^e^ Missing data for 2 participants.

^f^ Missing data for 14 participants.

^g^ Missing data for 13 participants.

^h^ Missing data for 6 participants.

^i^ Missing data for 7 participants.

^j^ Missing data for 8 participants.

^k^ Missing data for 10 participants.

**TABLE 2:** Dichotic listening characteristics of participant groups.

	Controls (*n* = 29)	tAD (*n* = 18)	lvPPA (*n* = 14)	nfvPPA (*n* = 10)	svPPA (*n* = 12)	rtvFTD (*n* = 6)	Significance testing results
VDLT total (/80)	76.34 (3.37)	**58.17 (13.77)** ^a^	**45.38 (12.06)** ^a^, ^b^	**47.60 (13.77)**	62.17 (16.26)^a^	74.17 (3.87)^a^	Overall model: F(9,78) = 17.69, *p* < 0.001 MMSE: F(1,78) = 24.84, *p* < 0.001 Diagnosis: F(5,78) = 8.77, *p* < 0.001 tAD vs Controls: *t* = −2.40, *p* = 0.019 lvPPA vs Controls: *t* = −2.45, *p* = 0.017 nfvPPA vs Controls: *t* = −6.37, *p* < 0.001 nfvPPA vs tAD: *t* = −3.91, *p* < 0.001 nfvPPA vs lvPPA: *t* = −2.26, *p* = 0.027 nfvPPA vs svPPA: *t* = −4.11, *p* < 0.001 nfvPPA vs rtvFTD: *t* = −4.57, *p* < 0.001 lvPPA vs rtvFTD: *t* = −2.05, *p* = 0.044
VDLT REA	*1.79 (2.34)*	*11.11 (9.30)*	*16.00 (11.05)*	*12.00 (18.56)*	7.67 (15.77)	*4.83 (3.66)*	Overall model: F(9,78) = 2.73, *p* = 0.008 *Control: t(28) = 4.13*, *p* < *0.001* *tAD: t(17) = 5.07, p* < *0.001* *lvPPA: t(12) = 5.22, p* < *0.001* *nfvPPA: t(9) = 2.04, p* = *0.036* *rtvFTD: t(5) = 3.80, p* = *0.006*
NVDLT total (/144)	135.52 (6.82)	**89.11 (19.65)** ^b^	**67.00 (17.61)** ^b^	**93.90 (26.13)** ^b^	**99.92 (36.46)**	123.67 (18.97)	Overall model: F(9,79) = 37.63, *p* < 0.001 MMSE: F(1,79) = 51.96, *p* < 0.001 PTA BEA: F(1,79) = 6.93, *p* = 0.01 Age: F(1,79) = 4.88, *p* = 0.03 Diagnosis: F(5,79) = 6.05, *p* < 0.001 tAD vs Controls: *t* = −4.03, *p* < 0.001 lvPPA vs Controls: *t* = −3.72, *p* < 0.001 nfvPPA vs Controls: *t* = −4.77, *p* < 0.001 svPPA vs Controls: *t* = −3.00, *p* = 0.004 tAD vs rtvFTD: *t* = −2.04, *p* = 0.045 lvPPA vs rtvFTD: *t* = −2.49, *p* = 0.015 nfvPPA vs rtvFTD: *t* = −2.67, *p* = 0.009
NVDLT LEA	0.28 (2.84)	0.56 (9.10)	−1.00 (15.10)	−*5.10 (8.02)*	1.58 (8.23)	4.00 (9.08)	Overall model: F (9,79) = 0.907, *p* = 0.54 *nfvPPA: t(9) =* −*2.01, p* = *0.038*
VDLT‐NVDLT difference (%)	1.32 (4.68)	10.83 (15.15)^a^	9.72 (19.25)^a^	−**5.71 (15.39)**	8.32 (19.76)^a^	6.83 (9.33)^a^	Overall model: F(9,78) = 2.82, *p* = 0.006 Diagnosis: F(5,78) = 2.12, *p* = 0.071 nfvPPA vs Controls: *t* = 2.21, *p* = 0.03 nfvPPA vs tAD: *t* = 2.86, *p* = 0.005 nfvPPA vs lvPPA: *t* = 2.05, *p* = 0.044 nfvPPA vs svPPA: *t* = 2.82, *p* = 0.006 nfvPPA vs rtvFTD: *t* = 2.18, *p* = 0.033

Mean (SD) values and raw scores are presented. Significant differences are indicated as follows: bold = significantly worse than healthy controls.

^a^ Significantly worse than nfvPPA.

^b^ Significantly worse than rtvFTD. *Italics* indicate a significant within‐subject right‐ear advantage for verbal dichotic listening or significant within‐participant left‐ear advantage for non‐verbal dichotic listening; positive values indicate a right‐ear advantage; negative values indicate a left‐ear advantage. The last column reports significant results from the analyses described in the main text (with the exception of the main effect of diagnosis for VDLT‐NVDLT difference score, which trended toward significance); see Table S2 for non‐significant results. Controls, healthy older volunteer group; LEA, left ear advantage; lvPPA, patient group with logopenic variant primary progressive aphasia (PPA); MMSE, Mini‐Mental State Examination; nfvPPA, patient group with nonfluent/agrammatic variant PPA; NVDLT, non‐verbal dichotic listening test; PTA BEA, pure‐tone audiometry better ear average; REA, right ear advantage; rtvFTD, patient group with right temporal variant frontotemporal dementia; svPPA, patient group with semantic variant PPA; tAD, patient group with typical Alzheimer's disease; VDLT, verbal dichotic listening test.

### 3.1 General participant group characteristics

Participant groups did not differ in years of education, handedness, symptom duration or peripheral hearing (all *p*’s > 0.05), but did differ in sex, age, and MMSE score (Table 1). All patient groups scored worse than the control group on real‐world hearing ability (indexed by mAIAD; Table 1).

### 3.2 Verbal dichotic listening

VDLT scores differed significantly across diagnostic groups (overall model: F(9, 78) = 17.69, *p* < 0.001; main effect of diagnosis: F(5, 78) = 8.77, *p* < 0.001) (Table 2, Figure 1). In between‐group comparisons, the control group performed significantly better than the groups with tAD (mean difference 8.52, 95% confidence interval [CI] 1.45–15.59), lvPPA (12.49, 2.33–22.66), and nfvPPA (24.13, 16.59–31.68). The nfvPPA group performed significantly worse than groups with tAD (−15.61, −23.57 to −7.66), lvPPA (−11.64, −21.90 to −1.38), svPPA (−18.85, −27.97 to −9.72), and rtvFTD (−23.76, −34.11 to −13.40), and the lvPPA group performed significantly worse than the rtvFTD group (12.12, 0.33–23.90) (Table 2, Figure 1).

One‐tailed *t*‐tests indicated a significant REA in controls (mean difference 1.79, 95% CI 0.90–2.68), tAD (11.11, 6.48–15.74), lvPPA (16.00, 9.32–22.68), nfvPPA (12.00, 8.49–27.11), and rtvFTD (6.33, 2.05–10.62), but not svPPA (7.67, −2.35 to 17.68) (Table 2; Table S2).

### 3.3 Non‐verbal dichotic listening performance

NVDLT scores also differed significantly across diagnostic groups (overall model: F(9,79) = 37.63, *p* < 0.001; main effect of diagnosis: F(5,79) = 6.05, *p* < 0.001 (Table 2, Figure 1). In between‐group comparisons, the control group performed significantly better than the groups with tAD (mean difference 21.45, 95% CI 10.86–32.04), lvPPA (28.16, 13.10–43.21), nfvPPA (27.10, 15.79–38.41), and svPPA (18.27, 6.16–30.38). The rtvFTD group performed significantly better than the groups with tAD (15.15, 0.35–29.95), lvPPA (21.86, 4.40–39.32), and nfvPPA (20.80, 5.28–36.31) (Table 2, Figure 1).

One‐tailed *t*‐tests indicated a significant LEA only in the nfvPPA group (mean difference −5.10, 95% CI −10.84 to 0.64) (Table 2).

For both the NVDLT and VDLT analyses, excluding participants with asymmetrical peripheral hearing impairment or digit span ≤4 did not substantially change the results (Supplementary Materials).

### 3.4 Comparison of verbal versus non‐verbal dichotic listening performance

For the difference between NVDLT and VDLT percentage scores, the overall model was significant (F(9,78) = 2.82, *p* = 0.006) and the effect of diagnosis trended toward significance (F(5,78) = 2.12, *p* = 0.071) (Table 2, Figure 1). In post hoc between‐group comparisons, the nfvPPA group showed disproportionate VDLT impairment, significantly different from all other groups (control mean difference: 11.40, 95% CI 1.11–21.70); tAD (15.57, 4.71–26.52); lvPPA (14.42, 0.42–28.42); svPPA (17.61, 5.17–30.06); and rtvFTD (15.44, 1.31–29.57) (Table 2, Figure 1).

### 3.5 Correlations of dichotic listening with other functions

Dichotic listening test scores were significantly correlated with each other, both within the control (*r = *0.46, *p* = 0.013) and combined patient (*r = *0.62, *p* < 0.001) cohorts (Figure S4).

PTA‐BEA was not significantly associated with VDLT performance in the control group (*r *= −0.35, *p* = 0.062) or combined patient cohort (*r *= −0.078, *p* = 0.56), but was significantly associated with NVDLT performance in both the control group (*r = *−0.55, *p* = 0.002) and combined patient cohort (*r *= −0.31, *p* = 0.016) (Figure S4).

Forward digit span was not significantly associated with either VDLT (*r *= −0.24, *p* = 0.21) or NVDLT (*r* = 0.07, *p* = 0.72) scores in controls, but significantly correlated both with VDLT (*r *= 0.62, *p* < 0.001) and NVDLT (*r* = 0.36, *p* = 0.004) scores in the combined patient cohort (Figure S4).

Total mAIAD score was more strongly correlated with NVDLT score than with MMSE, VDLT, or PTA‐BEA score (Figure 1E). Across mAIAD subscales, the strongest correlations were found for NVDLT score with subscales assessing sound detection and localization.

### 3.6 ROC analyses

The ROC analysis for discriminating all patients from controls on NVDLT revealed AUC 0.93 (95% CI 0.87–0.98), compared with AUCs 0.91 (95% CI 0.85–0.97) for VDLT, 0.90 (0.84–0.96) for MMSE score, and 0.61 (0.49–0.73) for PTA‐BEA (Figure 1F).

The ROC analysis for discriminating the tAD and control groups on NVDLT had AUC 0.99 (95% CI 0.99–1.0), compared with AUCs 0.95 (0.89–1.0) for VDLT, 0.98 (0.95–1.0) for MMSE score, and 0.66 (0.49–0.83) for PTA‐BEA (Figure 1D).

### 3.7 Neuroanatomic data

**FIGURE 2:**
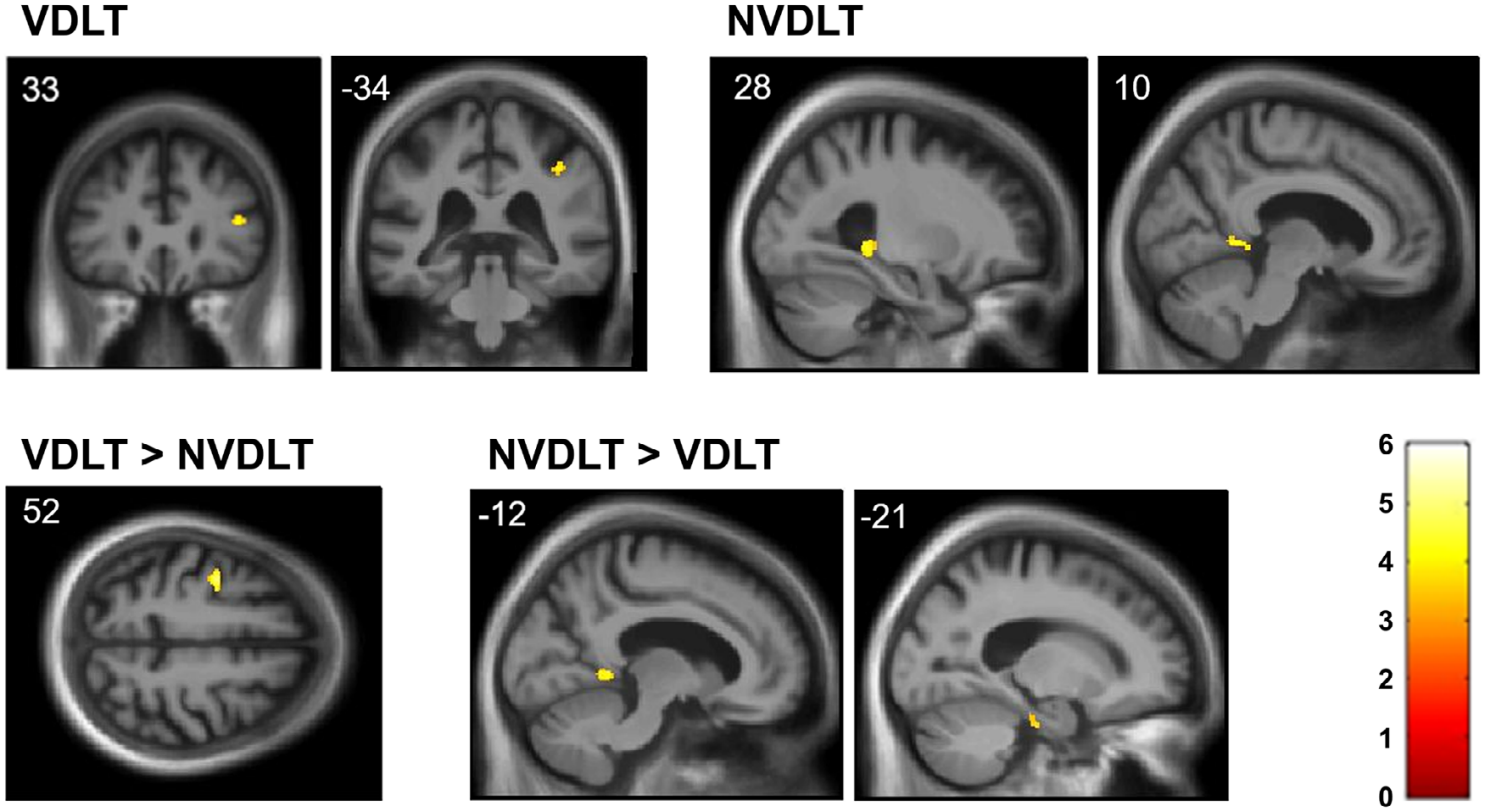
The VBM results. Statistical parametric maps of regional gray matter volume positively associated with performance on dichotic listening tests for the combined patient cohort. The top panels show gray matter correlates of verbal and non‐verbal dichotic listening test scores. The bottom panels show correlates of significant performance discrepancy between the dichotic conditions. Maps are rendered on sections of the group mean T1‐weighted brain MRI at *p*_FWE_ < 0.05 (see also Table S3) within prespecified neuroanatomic regions of interest (Figure S3) following an initial cluster‐defining threshold (*p* < 0.001). The left hemisphere is presented on the left for coronal sections and on top in the axial section; MNI coordinates for the plane of each section are indicated. The color bar codes voxel‐wise t scores for each map. Across the combined patient cohort, verbal dichotic listening performance was significantly associated with regional gray matter volume in the right inferior frontal gyrus and right supramarginal gyrus (both *p*_FWE_ < 0.05 after correction for multiple voxel‐wise comparisons within the relevant prespecified neuroanatomic region of interest). Non‐verbal dichotic listening performance was significantly associated with regional gray matter volume in the right posterior hippocampus and right retrosplenial cortex (both *p*_FWE_ < 0.05 after correction for multiple voxel‐wise comparisons within the relevant prespecified neuroanatomic region of interest). A left premotor cortical region was significantly more strongly associated with performance on the verbal dichotic listening test compared with the non‐verbal dichotic listening test; whereas the left retrosplenial and left entorhinal regions were significantly more strongly associated with performance on the non‐verbal dichotic test compared with the verbal test (all *p*_FWE_ < 0.05 after correction for multiple voxel‐wise comparisons within the relevant prespecified neuroanatomic region of interest). NVDLT, non‐verbal dichotic listening test; VDLT, verbal dichotic listening test.

Statistical parametric maps of gray matter regions associated with dichotic listening performance are shown in Figure 2, and local maxima are summarized in Table S3. At the prescribed significance threshold, VDLT score was correlated with gray matter in the right inferior frontal and supramarginal gyri and NVDLT score with gray matter in the right posterior hippocampus and retrosplenial cortex. Better performance on VDLT than NVDLT was correlated with gray matter in the left premotor cortex, and the converse with gray matter in the left retrosplenial and entorhinal cortices.

## 4 Discussion

Herein we report that both that non‐verbal and verbal dichotic listening are impaired in patients with tAD and PPA, after adjusting for peripheral hearing, age, sex, and MMSE score. For the NVDLT, all syndromic groups except rtvFTD performed worse than controls, and patients with tAD, lvPPA, and nfvPPA performed worse than those with rtvFTD. Consistently across mAIAD domains, daily‐life hearing function was more strongly correlated with NVDLT performance than with VDLT, PTA‐BEA, or MMSE scores: whereas the differential value of these tests in predicting everyday hearing was not quantified statistically, the findings support previous work suggesting that measures of auditory brain function may constitute a “real‐world audiogram” for dementia syndromes.^8^, ^29^

NVDLT score correlated with both PTA‐BEA and auditory working memory in the patient cohort. This underscores the complex interplay of these factors and the need for caution interpreting “central” hearing tests. Some of the non‐verbal sounds in the NVDLT include higher frequency ranges susceptible to the effects of presbycusis (Figure S1), whereas dichotic tests necessarily entail some working memory load. However, these factors do not entirely *account* for NVDLT performance profiles (NVDLT effects persisted after adjusting for PTA‐BEA and excluding patients with low working memory spans/asymmetric hearing impairment).

The tAD group had an elevated VDLT REA compared with controls, in accordance with previous findings.^10^ Only the nfvPPA group showed a NVDLT LEA: we did not find this in healthy controls as anticipated,^27^ perhaps reflecting stimulus factors or the effects of healthy aging. Further research should explore whether this is due to idiosyncrasies of individual stimuli or if LEA is attenuated with aging.

The neuroanatomic associations here suggest candidate mechanisms for syndromic DLT profiles. VDLT performance correlated with gray matter in the inferior frontal, supramarginal, and (more specifically, relative to non‐verbal dichotic processing) premotor areas previously implicated in processing competing and/or degraded speech signals.^15^, ^30^, ^31^, ^32^, ^33^, ^34^, ^35^, ^36^ Although interpretation should be cautious (because VDLT vs NVDLT comparisons within syndromic groups here were post hoc), these neuroanatomic correlates fit with the more selective VDLT impairment in the nfvPPA group. This syndrome targets peri‐Sylvian and prefrontal cortices, associated with impaired predictive decoding of acoustically challenging speech and other auditory signals.^32^, ^37^, ^38^ This mechanism may interact with impaired auditory temporal resolution to affect speech perception disproportionately in nfvPPA.^39^

NVDLT performance was correlated with gray matter in the hippocampus, entorhinal cortex, and retrosplenial cortex. The hippocampus is increasingly thought to play a key role in tracking and detecting auditory patterns/events,^14^, ^40^ whereas retrosplenial cortex has been implicated in ASA for non‐verbal sounds in tAD.^3^ Entorhinal cortex is implicated in cross‐modal auditory‐visual processing,^41^ a specific demand of the NVDLT. These areas are key components of the default mode network targeted by AD pathology, suggesting that the NVDLT may be a sensitive probe of tAD and its variants, particularly lvPPA. This is consistent with previous evidence that patients on the AD spectrum have particular difficulties disambiguating competing verbal and non‐verbal information.^2^, ^3^, ^8^, ^10^

In line with this, ROC analyses further suggest that the NVDLT would be an excellent clinical test for discriminating patients from healthy older individuals, and a near‐perfect clinical test for discriminating patients with tAD from healthy older individuals.^42^ Scores on the NVDLT here better predicted everyday hearing symptoms than did VDLT or PTA‐BEA, suggesting that this test may also serve as a useful proxy for daily‐life hearing function.

Our findings build on a growing body of work suggesting that measures of “brain hearing” (i.e. auditory cognition) are affected in the course of AD and other dementias.^1^ The potential applicability of the NVDLT across languages and cultures would help to fill an urgent clinical need for more inclusive and sensitive physiological tests to support dementia diagnosis and prediction of everyday communication function—with a view to treatment planning, and suitable for ethnically diverse older populations.^43^

This study has limitations. Regarding the construction of the NVDLT, future research should assess the impact of different sounds and sound combinations on test performance. For example, sounds with frequencies within the range of human speech would be more directly comparable to the VDLT and help assess potentially confounding effects from presbycusis. Further to the comparison between our NVDLT and the standard VDLT, it is noteworthy that these tests employed different response procedures (respectively, picture matching vs explicit digit recall, after hearing the sounds): future work could equate response modalities and working memory demands, to compare the tests more directly. Elucidation of the underlying neural mechanisms of non‐verbal (and verbal) dichotic listening in dementia syndromes will entail further structural and functional neuroimaging studies, with scope to assess syndrome‐specific as well as shared mechanisms of dichotic listening impairment.

Particularly in light of the heterogeneity of dementia syndromes, this work should be extended to larger cohorts representing linguistically and culturally diverse older populations, attending memory clinics beyond specialist centers. Although the everyday sounds employed in our NVDLT appear to be widely familiar across cultures,^27^ this requires direct substantiation. Larger cohorts would also support matching of patients to cognitively‐well controls by age, sex, and other demographic characteristics, as well as stratification on these factors and by disease severity. Longitudinal studies will also be important, to assess how changes in dichotic listening develop in relation to other clinical and biological markers of neurodegeneration.

## CONFLICTS OF INTEREST STATEMENT

The authors have no conflicts of interest to disclose. Any author disclosures are available in the Supporting Information.

## CONSENT STATEMENT

All participants provided written informed consent. Ethical approval was granted by the University College London ‐National Hospital for Neurology and Neurosurgery Joint Research Ethics Committee (06NO32), following Declaration of Helsinki guidelines.

## Supporting information



Supporting information

Supporting information
