# Supplementary material for: Non‐verbal dichotic listening: A new cognitive hearing test for dementia
Source: Alzheimers Dement. 2026 May 26;22(5):e71358. doi: 10.1002/alz.71358 (PMC13240222; doi:10.1002/alz.71358)
Supplement: Supplementary file 2 — Supporting information [file ALZ-22-e71358-s001.docx]

**SUPPLEMENTARY MATERIAL**

**Non-verbal dichotic listening: a new cognitive hearing test for dementia,**

by Chris JD Hardy, Benjamin A Levett et al

**Participants**

All patients fulfilled criteria^1–3^ for the relevant clinical syndrome.

No participant had a history of otological disease, other than presbycusis. Two tAD, one nfvPPA, one svPPA and one rtvFTD participant wore hearing aids (with headphones). All patients (21/21) with tAD and lvPPA who were assessed for AD biomarkers (cerebrospinal fluid ptau181 and beta-amyloid^42:40^ ratio or blood ptau-217) tested positive, based on local reference ranges.

Peripheral hearing and verbal dichotic listening data were previously published for a subset of eight patients with tAD, six with lvPPA, four with nfvPPA, eight with svPPA, and 14 healthy control participants ^4^.

One participant with logopenic variant primary progressive aphasia (lvPPA) was unable to complete the verbal dichotic listening task due to a lack of time, therefore leaving a total of 13 patients with lvPPA for any analyses involving verbal dichotic listening task score.

Five participants (one control, three lvPPA, one nfvPPA) were identified as having asymmetrical peripheral hearing impairment – we therefore ran parallel analyses excluding these participants (see below).

**Peripheral hearing assessment**

Following British Society of Audiology guidelines ^5^, pure-tone audiometry was performed at 250, 500, 1000, 2000, 4000 and 8000 Hz using either a dual-channel GSI Audiostar Pro audiometer or an Amplivox Screening audiometer model 116, with calibrated, noise-reducing headphones in a quiet room. The pure-tone threshold for each ear was calculated as the average minimum threshold in decibels hearing level (dB HL) across the 500, 1000, 2000 and 4000 Hz frequencies as these are most relevant to speech processing ^6^; the lowest of these was selected as the ‘better ear average’ (BEA) for each participant.

Participants were classified as having asymmetrical hearing loss if the difference for at least two adjacent frequencies between right and left ears was ≥20dB (2AF20^7^).

**Neuropsychological assessment**

A general neuropsychological battery was administered to participants alongside their dichotic listening assessments. The researcher ensured that all spoken instructions and practice trials were delivered at a sound level that was easily audible for each participant, and participants were allowed to wear hearing aids if preferred.

**Modified Amsterdam Inventory for Auditory Disability and Handicap**

To assess daily-life hearing function in the patient groups, each patient’s primary caregiver completed the modified Amsterdam Inventory for Auditory Disability and Handicap (mAIAD)^8^. Control participants completed the questionnaire themselves.

The scale contains 28 items probing domains of auditory detection, auditory localisation, speech-in-noise, speech-in-quiet and auditory discrimination. On each item, the respondent is presented with a Likert scale with response options of “Almost always” (4 points), “Frequently” (3 points), “Occasionally” (2 points), and “Almost never” (1 point). All items assess the person’s ability to hear in a specific situation, so higher scores indicate better hearing.

**Verbal dichotic listening assessment**

Verbal dichotic listening stimuli were administered from a Dell Latitude laptop computer and using Audio-Technica M50x headphones in a quiet room. Prior to commencing the task, participants were played a 1kHz test tone, asked to confirm that they could hear this tone in both ears, and invited to adjust the volume to a comfortable listening level (at least 70dB). There were no practice trials for the verbal dichotic listening task (VDLT). One participant with lvPPA had only completed the NVDLT meaning that all analyses which included VDLT score in the model did not include this patient. Twenty trials were administered; the maximum score was 4 on each trial and 80 in toto.

**Non-verbal dichotic listening test development**

Soundfiles for the non-verbal dichotic listening task (NVDLT) were selected from a previously published corpus ^9^. From this database, we chose sounds that a) were not animal noises; b) were not likely to be idiosyncratic to a particular location (e.g. dial tone/ ambulance siren); c) were relatively continuous for a two-second period (i.e. not an intermittent sound like hammering) and did not depend on a small part of the sound for identification (e.g. the ‘ping’ of a microwave); d) were not musical instruments; and e) did not contain speech. This resulted in a subset of 17 possible sounds which were piloted with five younger healthy volunteers (all female; average age 28.2 years, 3.8 years standard deviation) who were asked to each sound, name it and give a rating of their confidence in identifying the sound correctly, and their familiarity with the sound (Table S1). The nine sounds (baby babbling, typing, gargling, whistle, laughing, groaning, bell, pouring liquid and thunder) which were consistently identified by the pilot participants and with the highest confidence ratings were selected as the final set of individual sounds for generating the dichotic trials with (with nine identified as the optimum number to match to the number of digits in the verbal version of the task, i.e. 1 through 10, with 7 omitted); see Figure S2. Four sounds (electric drill, brushing teeth, ticking clock, washing dishes) that were not included in this final set of nine were selected for use in practice trials (Figure S2).

Pairs of soundfiles for both the main and experimental tasks were generated in Audacity v 3.2.0 with one sound assigned to the left channel at the same onset time as a different sound was assigned to the right channel. This process generated six unique sound pairings for the practice trial and 36 unique sound pairings for the experimental trials. For the practice trials, just one sound pair was presented, with the sounds overlapping such that one sound was presented to the left ear and the other simultaneously to the right ear. For the main non-verbal dichotic task, consistent with the verbal dichotic task, a 0.5 second gap was added after the first sound pair and a second sound pairing then followed. Sound pairings were combined pseudo-randomly across trials such that no sound was ever repeated within trial, creating 18 trials which were then duplicated with channels switched to generate a total of 36 trials. The trial order was pseudo-randomised such that a maximum of one sound was repeated in the immediately subsequent trial. Average (rms) stimulus intensity was constant for all stimuli, as fixed in Matlab. All stimuli were windowed with 20ms onset-offset temporal ramps to prevent click artefacts. All stimuli were administered from a Dell Latitude laptop computer and using Audio-Technica M50x headphones in a quiet room.

**Non-verbal dichotic listening assessment**

During the practice phase, two sounds were played on each trial with the sounds overlapping such that one sound was presented to the left ear and the other to the right ear. After each practice trial, the participant was asked to identify the sounds that they heard, in any order, using the response matrix provided (Figure S2A). Four trials were administered, with the participant proceeding to the main task if they were able to identify at least one sound correctly across the four trials.

For the main NVDLT, prior to commencing the task proper, the participant was first played each of the nine possible sounds and asked to identify the sound from the response matrix (Figure S2B). Participants were allowed to ask for sounds to be repeated as many times as they liked but were only able to proceed to the main task when they had reliably matched each sound to the corresponding representation in the visual response matrix. One participant with Alzheimer’s disease was excluded as they were not able to do this. Thirty-six trials were administered; the maximum score was 4 on each trial and 144 in toto.

**Ear advantages**

Previous research has identified lateralised ear advantages associated with different types of auditory stimuli (right ear advantage (REA) for verbal material; left ear advantage (LEA) for non-verbal material^10,11^). Therefore, for VDLT we calculated a REA score by taking total number of digits recalled from stimuli presented to the left ear away from digits recalled from stimuli presented to the right ear. For NVDLT to calculate a LEA score, we did the analogous procedure for the nonverbal sound stimuli. We then conducted within-subjects one-tailed one-sample t-tests to assess whether the ear advantage was significantly above zero (for VDLT, indicating a significant REA) or below zero (for NVDLT, indicating a significant LEA) in each group. Second, we conducted an ANCOVA model, as described above, to assess whether there was a between-subjects effect of diagnosis on ear advantage.

**Brain image acquisition and analysis**

Brain images were acquired in a 3T Siemens Prisma MRI scanner using a 64-channel phased array head coil and following a T1-weighted sagittal 3D magnetization prepared rapid gradient echo (MPRAGE) sequence (echo time = 2.9 ms, inversion time = 900 ms, repetition time = 2200 ms), with dimensions 256 mm × 256 mm × 208 mm and voxel size 1.1 mm × 1.1 mm × 1.1 mm.

For the voxel-based morphometry (VBM) analysis, patients’ brain images were first pre-processed and normalised to MNI space using Statistical and Parametric Mapping (SPM) software v25 and the SHOOT toolbox with default parameters running under MATLAB R2023b. Images were smoothed using a 6mm full width at half-maximum (FWHM) Gaussian kernel. Total intracranial volume was calculated for each patient by summing white matter, grey matter and CSF volumes post-segmentation ^12^, in order to control for individual differences in pre-morbid brain size. We used an automatic mask-creating strategy to create an explicit, study-specific brain mask ^13^. A study-specific mean brain template image was created by warping all patients’ native-space whole-brain images to the final SHOOT template and using the ImCalc function to generate an average of these images.

We assessed grey matter associations of verbal and non-verbal dichotic listening over the combined patient cohort in a multiple regression design incorporating the covariates of dummy-coded diagnostic group membership, age, sex, total intracranial volume, MMSE score, and PTA better ear average score. We used contrast vectors of [1 -1] and [-1 1] to identify regions where grey matter intensity was more strongly associated with performance on the VDLT vs NVDLT, and vice versa. Statistical parametric maps were generated using an initial cluster-defining threshold (p < 0.001) and assessed at peak-level significance threshold p<0.05 after family-wise error (FWE) correction for multiple voxel-wise comparisons within seven separate predefined regions of interest, based on previously published work^4,14–23^ (see Figure S3).

**Supplementary analyses excluding patients with most severe working memory deficits or asymmetrical hearing loss**

***Verbal dichotic listening performance***

Excluding participants with asymmetrical peripheral hearing loss from the analyses did not substantially change the results as reported in the main paper: the overall model remained significant (F(9,73)=18.79, p<0.001). MMSE (F(1,73)=30.00, p<0.001) and diagnosis (F(5,73)=7.91, p<0.001) were significantly associated with verbal dichotic listening performance whilst the other covariates were not (p>0.05). Post-hoc t-tests showed that the healthy control group performed significantly better than the patient groups with tAD (covariate-adjusted mean difference: -7.52 [95%CI -14.53 to -0.53], t=-2.14, p=0.035), lvPPA (-12.50 [95%CI -23.21 to -1.80], t=-2.33, p=0.023) and nfvPPA (-23.19 [95%CI -30.95 to -15.43], t=-6.37, p<0.001). The nfvPPA group again performed significantly worse than all other patient groups (vs tAD adjusted mean: -15.65 [95%CI -23.61 to -7.72], t=-3.93, p<0.001; vs lvPPA: -10.69 [95%CI -21.27 to -0.11], t=-2.01, p=0.048; vs svPPA: -18.65 [95%CI -27.61 to -9.67], t=-4.14, p<0.001; vs rtvFTD: -23.26 [95%CI -33.46 to -13.06], t=-4.54, p<0.001), while the rtvFTD group again performed significantly better than the lvPPA group (adjusted mean difference: -12.57 [95%CI -24.48 to -0.66], t=-2.10, p=0.039). No other between-group comparisons were significant (all p>0.05).

Excluding participants with digit span ≤4 from the analyses did not substantially change the results as reported in the main paper: the overall model remained significant (F(9,72) = 14.96, p < 0.001) and MMSE score (F(1,72) = 24.99, p < 0.001) and diagnosis (F(5,72) = 8.18, p < 0.003) both remained significant, with no significant effects of age, sex or pure-tone audiometry better ear average.

Patient groups with tAD and nfvPPA were again significantly different from the healthy control group (vs tAD covariate-adjusted mean difference: -7.67 [95%CI -14.36 to -0.97], t = -2.28, p = 0.026; vs nfvPPA: -22.52 [95%CI -29.92 to -15.13], t = -6.07, p < 0.001). The nfvPPA group were again significantly more impaired than each other patient group (vs tAD: -14.86 [95%CI -22.64 to -7.08], t = -3.81, p < 0.001; vs lvPPA: -17.13 [95%CI -27.88 to -6.38], t = -3.18, p = 0.002; vs svPPA: -16.97 [95%CI -25.82 to -8.13], t = -3.83, p < 0.001; rtvFTD: -22.50 [95%CI -32.33 to -12.66], t = -4.56, p < 0.001). The difference between the rtvFTD and lvPPA group, however, was no longer significant.

***Non-verbal dichotic listening performance***

Excluding participants with asymmetrical peripheral hearing loss from the analyses did not substantially change the results as reported in the main paper: the overall model was significant (F(9,74)=37.54, p<0.001). MMSE (F(1,74)=56.76, p<0.001), PTA-BEA (F(1,74)=8.09, p=0.006) and diagnosis (F(5,74)=4.67, p<0.001) were significantly associated with NVDLT score whilst sex was not (p>0.05). The association with age was no longer significant (F(1,74) = 3.78, p = 0.056). Post-hoc t-tests showed that all patient groups except rtvFTD performed significantly worse than the healthy controls (adjusted-mean difference vs tAD: -20.57 [95%CI -31.13 to -10.00], t=-3.88, p<0.001); vs lvPPA: -28.12 [95%CI -44.01 to -12.24], t=-3.53, p<0.001; vs nfvPPA: -22.74 [95%CI -34.45 to -11.03], t=-3.87, p<0.001; vs svPPA: -16.78 [95%CI -28.85 to -4.71], t=-2.77, p=0.007. The rtvFTD group also performed significantly better on average than the tAD (adjusted mean difference -15.08 [95%CI -29.57 to -0.60], t=-2.07, p=0.042), lvPPA (-22.64 [95%CI -40.32 to -4.96], t=-2.55, p=0.013) and nfvPPA (-17.25 [95%CI -32.64 to -1.87], t=-2.23, p=0.028) groups. No other between-group comparisons were significant (all p>0.05).

Excluding participants with digit span ≤4 from the analyses did not substantially change the results as reported in the main paper: the overall model remained significant (F(9,73) = 34.03, p < 0.001). MMSE (F(1,73) = 42.73, p < 0.001), PTA-BEA (F(1,73) = 4.83, p = 0.031), age (F(1,73) = 7.34, p = 0.008) and diagnosis (F(5,73) = 6.29, p < 0.001) were significantly associated with non-verbal dichotic listening performance whilst sex was not (p > 0.05). Post-hoc tests again showed that all patient groups with exception of the rtvFTD group (covariate-adjusted mean difference -7.41 [95%CI -21.35 to 6.52], t = -1.06, p = 0.293) performed significantly worse than the healthy controls (adjusted-mean difference vs tAD: -21.98 [95%CI -32.67 to -11.30], t = -4.10, p < 0.001); vs lvPPA: -30.00 [95%CI -46.79 to -13.22], t = -3.56, p = 0.001; vs nfvPPA: -28.75 [95%CI -40.55 to -16.96], t = -4.86, p < 0.001; vs svPPA: -20.36 [95%CI -32.80 to -7.92], t = -3.26, p = 0.002). The rtvFTD group also performed significantly better on average than the lvPPA (- 22.59 [95%CI -41.17 to -4.01], t = -2.42, p = 0.018) and nfvPPA (-21.34 [95%CI -37.03 to -5.66], t = -2.71, p = 0.008) groups, but the comparison with the tAD group was no longer significant.

***Comparison of verbal vs non-verbal dichotic listening performance***

Excluding participants with asymmetrical peripheral hearing loss from the analyses did not substantially change the results as reported in the main paper: the overall model was significant (F(9,73)=2.74, p=0.008). Unlike in the main model, excluding these five participants resulted in a significant main effect of diagnosis (F(5,73)=2.61, p=0.032; no other covariates were significant (all p>0.05). Post-hoc tests showed that the nfvPPA group scored significantly differently from all participant groups on this measure, reflecting disproportionate impairment on VDLT (adjusted mean difference vs healthy controls: 13.24 [95%CI 2.44 to 24.03], t=2.44, p=0.017; vs tAD: 18.03 [95%CI 6.98 to 29.08], t=3.25, p=0.002; vs lvPPA: 16.15 [95%CI 1.42 to 30.87], t=2.19, p=0.032; vs svPPA: 19.30 [95%CI 6.81 to 31.78], t=3.08, p=0.003]; vs rtvFTD: 17.22 [95%CI 3.02 to 31.41], t=2.42, p=0.018). No other between-group comparisons were significant (all p>0.05).

Excluding participants with digit span ≤4 from the analyses did not substantially change the results as reported in the main paper: the overall model remained significant (F(9,72) = 3.48, p = 0.001 and the effect of diagnosis again trended toward significance (F(5,72) = 2.15, p = 0.070); no other covariates were significant (all p > 0.01). Post-hoc tests showed that the nfvPPA group scored significantly differently from all other patient groups on this measure due to their disproportionate deficit on the verbal dichotic listening test (adjusted mean difference vs tAD: 13.81 [95%CI 3.33 to 24.28], t = 2.63, p = 0.011; vs lvPPA: 20.80 [95%CI 6.33 to 35.27], t = 2.86, p = 0.005; vs svPPA: 15.64 [95%CI 3.73 to 27.56], t = 2.62, p = 0.011]; vs rtvFTD: 13.52 [95%CI 0.28 to 26.77], t = 2.04, p = 0.045) (Figure 1; Figure S3); however, the nfvPPA vs healthy control comparison was no longer significant. No other between-group comparisons were significant (all p > 0.05).

**Supplementary references**

1. Dubois B, Feldman HH, Jacova C, et al. Advancing research diagnostic criteria for Alzheimer’s disease: the IWG-2 criteria. *The Lancet Neurology*. 2014;13(6):614-629. doi:10.1016/S1474-4422(14)70090-0

2. Gorno-Tempini ML, Hillis AE, Weintraub S, et al. Classification of primary progressive aphasia and its variants. *Neurology*. 2011;76(11):1006-1014. doi:10.1212/WNL.0b013e31821103e6

3. Ulugut H, Bertoux M, Younes K, et al. Clinical recognition of frontotemporal dementia with right anterior temporal predominance: A multicenter retrospective cohort study. *Alzheimer’s & Dementia*. 2024;20(8):5647-5661. doi:10.1002/alz.14076

4. Jiang J, Johnson J, Levett BA, et al. Pure-tone audiometry and dichotic listening in primary progressive aphasia and Alzheimer’s disease. *Quarterly Journal of Experimental Psychology*. Published online September 19, 2024:17470218241287349. doi:10.1177/17470218241287349

5. BSA BS of A. *Recommended Procedure Pure-Tone Air-Conduction and Bone-Conduction Threshold Audiometry with and without Masking*.; 2018. Accessed August 20, 2018. www.thebsa.org

6. Lin FR, Reed NS. The Pure-Tone Average as a Universal Metric—Knowing Your Hearing. *JAMA Otolaryngology–Head & Neck Surgery*. 2021;147(3):230-231. doi:10.1001/jamaoto.2020.4862

7. British Academy of Audiology. 2016. Guidance for Audiologists: Onward Referral of Adults With Hearing Difficulty Directly Referred to Audiology Services. Reading, UK: British Academy of Audiology. - Google Search. Accessed October 16, 2025. https://www.google.com/search?q=British+Academy+of+Audiology.+2016.+Guidance+for+Audiologists%3A+Onward+Referral+of+Adults+With+Hearing+Difficulty+Directly+Referred+to+Audiology+Services.+Reading%2C+UK%3A+British+Academy+of+Audiology.&rlz=1C1CHBF_en-GBGB990GB990&oq=British+Academy+of+Audiology.+2016.+Guidance+for+Audiologists%3A+Onward+Referral+of+Adults+With+Hearing+Difficulty+Directly+Referred+to+Audiology+Services.+Reading%2C+UK%3A+British+Academy+of+Audiology.&gs_lcrp=EgZjaHJvbWUyBggAEEUYOTIGCAEQRRg80gEHNTk3ajBqNKgCALACAQ&sourceid=chrome&ie=UTF-8

8. Meijer AGW, Wit HP, TenVergert EM, Albers FWJ, Muller Kobold JEP. Reliability and validity of the (modified) Amsterdam Inventory for Auditory Disability and Handicap. *Int J Audiol*. 2003;42(4):220-226. doi:10.3109/14992020309101317

9. Norman-Haignere S, Kanwisher NG, McDermott JH. Distinct Cortical Pathways for Music and Speech Revealed by Hypothesis-Free Voxel Decomposition. *Neuron*. 2015;88(6):1281-1296. doi:10.1016/j.neuron.2015.11.035

10. Palaniswamy HP, Bhat M, Bhat RG, Krishna Y, Rajashekhar B. Development, and validation of non-speech dichotic listening test. *J Otol*. 2023;18(1):63-69. doi:10.1016/j.joto.2022.12.004

11. Kimura D. Cerebral dominance and the perception of verbal stimuli. *Canadian Journal of Psychology / Revue canadienne de psychologie*. 1961;15(3):166-171. doi:10.1037/h0083219

12. Malone IB, Leung KK, Clegg S, et al. Accurate automatic estimation of total intracranial volume: a nuisance variable with less nuisance. *NeuroImage*. 2015;104:366-372. doi:10.1016/j.neuroimage.2014.09.034

13. Ridgway GR, Omar R, Ourselin S, Hill DLG, Warren JD, Fox NC. Issues with threshold masking in voxel-based morphometry of atrophied brains. *NeuroImage*. 2009;44(1):99-111. doi:10.1016/j.neuroimage.2008.08.045

14. Billig AJ, Lad M, Sedley W, Griffiths TD. The hearing hippocampus. *Prog Neurobiol*. 2022;218:102326. doi:10.1016/j.pneurobio.2022.102326

15. Golden HL, Agustus JL, Goll JC, et al. Functional neuroanatomy of auditory scene analysis in Alzheimer’s disease. *NeuroImage: Clinical*. 2015;7:699-708. doi:10.1016/J.NICL.2015.02.019

16. Goll JC, Kim LG, Ridgway GR, et al. Impairments of auditory scene analysis in Alzheimer’s disease. *Brain : a journal of neurology*. 2012;135(Pt 1):190-200. doi:10.1093/brain/awr260

17. Hardy CJD, Marshall CR, Bond RL, et al. Retained capacity for perceptual learning of degraded speech in primary progressive aphasia and Alzheimer’s disease. *Alzheimer’s Research & Therapy*. 2018;10(1):70. doi:10.1186/s13195-018-0399-2

18. Jäncke L, Buchanan TW, Lutz K, Shah NJ. Focused and Nonfocused Attention in Verbal and Emotional Dichotic Listening: An FMRI Study. *Brain and Language*. 2001;78(3):349-363. doi:10.1006/brln.2000.2476

19. Jiang J, Johnson JCS, Requena-Komuro MC, et al. Comprehension of acoustically degraded speech in Alzheimer’s disease and primary progressive aphasia. *Brain*. Published online May 15, 2023:awad163. doi:10.1093/brain/awad163

20. Johnson JCS, Jiang J, Bond RL, et al. Impaired phonemic discrimination in logopenic variant primary progressive aphasia. *Annals of Clinical and Translational Neurology*. 2020;7(7):1252-1257. doi:10.1002/acn3.51101

21. Kaestner E, Wu X, Friedman D, et al. The Precentral Gyrus Contributions to the Early Time-Course of Grapheme-to-Phoneme Conversion. *Neurobiology of Language*. 2022;3(1):18-45. doi:10.1162/nol_a_00047

22. Schubotz RI, von Cramon DY, Lohmann G. Auditory what, where, and when: a sensory somatotopy in lateral premotor cortex. *NeuroImage*. 2003;20(1):173-185. doi:10.1016/S1053-8119(03)00218-0

23. Thomsen T, Rimol LM, Ersland L, Hugdahl K. Dichotic listening reveals functional specificity in prefrontal cortex: an fMRI study. *NeuroImage*. 2004;21(1):211-218. doi:10.1016/j.neuroimage.2003.08.039

**SUPPLEMENTARY TABLES AND FIGURES**

**Table S1.** Piloting of soundfiles for the non-verbal dichotic stimuli

| **Sound** | **Successful identification** | **Confidence** | **Familiarity** |
| --- | --- | --- | --- |
| Baby babbling | 1 | 10 | 8.8 |
| Typing | 1 | 9.8 | 9.8 |
| Gargling | 1 | 9.8 | 9.4 |
| Whistle | 1 | 9.8 | 9.2 |
| Laughing | 1 | 9.6 | 9.8 |
| Grunting/groaning | 1 | 9.6 | 9.6 |
| School bell | 1 | 9.6 | 9.4 |
| Pouring water | 1 | 8.8 | 9.4 |
| Thunder | 1 | 8.8 | 8.8 |
| Running water | 1 | 8.6 | 9.2 |
| Electric hand drill | 1 | 8.2 | 8.4 |
| Tooth-brushing | 1 | 8 | 9.2 |
| Water dripping | 1 | 7.6 | 8.6 |
| Dishes clanking | 0.8 | 9 | 9.6 |
| Ticking clock | 0.8 | 7.4 | 7.2 |
| Flapping wings | 0.6 | 3.6 | 6.2 |
| Tearing | 0.4 | 6.6 | 7.4 |

Piloting results from five healthy younger volunteers are shown; see Non-verbal dichotic test development section above for more information.

**Table S2.** Dichotic listening characteristics of participant groups

|  | **Controls** | **tAD** | **lvPPA** | **nfvPPA** | **svPPA** | **rtvFTD** | **Significance testing results** |
| --- | --- | --- | --- | --- | --- | --- | --- |
| VDLT Total (/80) | 76.34 (3.37) | **58.17 (13.77)^a^** | **45.38 (12.06)^a,b^** | **47.60 (13.77)** | 62.17 (16.26)^a^ | 74.17 (3.87)^a^ | Overall model: F(9,78)=17.69, p<0.001  Age: F(1,78)=0.09, p=0.77  Sex: F(1,78)=0.02, p=0.89  PTA-BEA: F(1,78)=0.12, p=0.73  MMSE: F(1,78)=24.84, p<0.001  Diagnosis: F(5,78)=8.77, p<0.001  tAD vs Controls: t=-2.40, p=0.019  lvPPA vs Controls: t=-2.45, p=0.017  nfvPPA vs Controls: t=-6.37, p<0.001  nfvPPA vs tAD: t=-3.91, p<0.001  nfvPPA vs lvPPA: t=-2.26, p=0.027  nfvPPA vs svPPA: t=-4.11, p<0.001  nfvPPA vs rtvFTD: t=-4.57, p<0.001  lvPPA vs rtvFTD: t=-2.05, p=0.044  Controls vs svPPA: t=-1.30, p=0.20  Controls vs rtvFTD: t=-0.08, p=0.94  tAD vs lvPPA: t=-0.96, p=0.34  tAD vs svPPA: t=0.83, p=0.41  tAD vs rtvFTD: t=1.64, p=0.11  lvPPA vs svPPA: t=1.62, p=0.11  svPPA vs rtvFTD: t=0.95, p=0.35 |
| VDLT REA | *1.79 (2.34)* | *11.11 (9.30)* | *16.00 (11.05)* | *12.00 (18.56)* | 7.67 (15.77) | *4.83 (3.66)* | Overall model: F(9,78)=2.73, p=0.008  Age: F(1,78)=1.14, p=0.29  Sex: F(1,78)=0.12, p=0.73  PTA-BEA: F(1,78)=0.40, p=0.53  MMSE: F(1,78)=0.29, p=0.59  Diagnosis: F(5,78)=1.44, p=0.22  *Control: t(28)=4.13, p<0.001*  *tAD: t(17)=5.07, p<0.001*  *lvPPA: t(12)=5.22, p<0.001*  *nfvPPA: t(9)=2.04, p=0.036*  *rtvFTD: t(5)=3.80, p=0.006*  *svPPA: t(11)=1.68, p=0.06* |
| NVDLT Total (/144) | 135.52 (6.82) | **89.11 (19.65)^b^** | **67.00 (17.61)^b^** | **93.90 (26.13)^b^** | **99.92 (36.46)** | 123.67 (18.97) | Overall model: F(9,79)=37.63, p<0.001  MMSE: F(1,79)=51.96, p<0.001  PTA-BEA: F(1,79)=6.93, p=0.01  Age: F(1,79)=4.88, p=0.03  Diagnosis: F(5,79)=6.05, p<0.001  Sex: F(1,79)=0.01, p=0.92  tAD vs Controls: t=-4.03, p<0.001  lvPPA vs Controls: t=-3.72, p<0.001  nfvPPA vs Controls: t=-4.77, p<0.001  svPPA vs Controls: t=-3.00, p=0.004  tAD vs rtvFTD: t=-2.04, p=0.045  lvPPA vs rtvFTD: t=-2.49, p=0.015  nfvPPA vs rtvFTD: t=-2.67, p=0.009  Controls vs rtvFTD: t=-0.90, p=0.37  tAD vs lvPPA: t=-1.09, p=0.28  tAD vs nfvPPA: t=-0.94, p=0.35  tAD vs svPPA: t=0.54, p=0.59  lvPPA vs nfvPPA: t=0.14, p=0.89  lvPPA vs svPPA: t=1.52, p=0.13  nfvPPA vs svPPA: t=1.29, p=0.20  svPPA vs rtvFTD: t=1.54, p=0.13 |
| NVDLT LEA | 0.28 (2.84) | 0.56 (9.10) | -1.00 (15.10) | *-5.10 (8.02)* | 1.58 (8.23) | 4.00 (9.08) | Overall model: F (9,79)=0.907, p=0.54  Age: F(1,79)=0.25, p=0.62  Sex: F(1,79)=0.59, p=0.44  PTA-BEA: F(1,79)=0.01, p=0.92  MMSE: F(1,79)=1.95, p=0.17  Diagnosis: F(5,79)=0.94, p=0.46  *nfvPPA: t(9)=-2.01, p=0.038*  *Control: t(28)=0.52, p=0.70*  *tAD: t(17)=0.26, p=0.60*  *lvPPA: t(13)=-0.25, p=0.40*  *svPPA: t(11)=0.67, p=0.74*  *rtvFTD: t(5)=1.08, p=0.84* |
| VDLT-NVDLT Difference (%) | 1.32 (4.68) | 10.83 (15.15)^a^ | 9.72 (19.25)^a^ | **-5.71 (15.39)** | 8.32 (19.76)^a^ | 6.83 (9.33)^a^ | Overall model: F(9,78)=2.82, p=0.006  Diagnosis: F(5,78)=2.12, p=0.071  Age: F(1,78)=2.31, p=0.13  Sex: F(1,78)=0.00, p=0.99  PTA-BEA: F(1,78)=2.57, p=0.11  MMSE: F(1,78)=0.93, p=0.34  nfvPPA vs Controls: t=2.21, p=0.03  nfvPPA vs tAD: t=2.86, p=0.005  nfvPPA vs lvPPA: t=2.05, p=0.044  nfvPPA vs svPPA: t=2.82, p=0.006  nfvPPA vs rtvFTD: t=2.18, p=0.033  Controls vs tAD: t=0.86, p=0.39  Controls vs lvPPA: t=0.43, p=0.67  Controls vs svPPA: t=1.12, p=0.27  Controls vs rtvFTD: t=0.64, p=0.53  tAD vs lvPPA: t=0.20, p=0.84  tAD vs svPPA: t=0.38, p=0.70  tAD vs rtvFTD: t=0.02, p=0.99  lvPPA vs svPPA: t=0.53, p=0.60  lvPPA vs rtvFTD: t=0.13, p=0.90  svPPA vs rtvFTD: t=0.31, p=0.76 |

Mean (standard deviation) values and raw scores are presented. Significant differences are indicated as follows: **bold** = significantly worse than healthy controls; ^a^significantly worse than nfvPPA; ^b^significantly worse than rtvFTD. *Italics* indicate a significant within-subjects right-ear advantage for verbal dichotic listening or significant within-participant left-ear advantage for non-verbal dichotic listening (see text); positive values indicate a right-ear advantage; negative values indicate a left-ear advantage. The Significance testing results column reports all results (whether significant [black font] or non-significant [underlined font]) from the analyses described in the main text and in Table 2. An overall model for VDLT REA comparing across diagnostic groups adjusting for covariates was significant (F(9,78)=2.73, p=0.008), but the main effect of diagnosis was not significant (p=0.218). An overall model for NVDLT LEA score comparing across diagnostic groups adjusting for covariates was not significant (F(9,79)=0.907, p=0.54). Controls, healthy control group; LEA, left ear advantage; lvPPA, patient group with logopenic variant primary progressive aphasia; MMSE, Mini-Mental State Examination; nfvPPA, patient group with nonfluent/agrammatic variant primary progressive aphasia; NVDLT, non-verbal dichotic listening test; PTA-BEA, pure-tone audiometry better ear average; REA, right ear advantage; rtvFTD, patient group with right temporal variant frontotemporal dementia; svPPA, patient group with semantic variant primary progressive aphasia; tAD, patient group with typical Alzheimer’s disease; VDLT, verbal dichotic listening test.

**Table S3.** Neuroanatomical associations of dichotic listening performance in the combined patient cohort

| **Region** | **Peak (mm)** | | | **T score** | **P_FWE_** |
| --- | --- | --- | --- | --- | --- |
|  | **x** | **y** | **z** |  |  |
| **Verbal dichotic listening** | | | | | |
| Right inferior frontal gyrus | 44 | 33 | 14 | 4.04 | 0.026 |
| Right supramarginal gyrus | 40 | -34 | 44 | 4.09 | 0.037 |
| **Non-verbal dichotic listening** | | | | | |
| Right posterior hippocampus | 28 | -36 | -2 | 4.20 | 0.011 |
| Right retrosplenial cortex | 10 | -48 | 2 | 4.25 | 0.042 |
| **Verbal > non-verbal dichotic listening** | | | | | |
| Left premotor cortex | -36 | -4 | 52 | 4.99 | 0.007 |
| **Non-verbal > verbal dichotic listening** | | | | | |
| Left retrosplenial cortex | -12 | -48 | 0 | 4.44 | 0.026 |
| Left entorhinal cortex | -21 | -21 | -30 | 3.84 | 0.041 |

The table shows significant positive associations between regional grey matter volume and dichotic listening performance, based on the voxel-based morphometric analysis of brain MR images for the combined patient cohort. X, y and z coordinates of peaks (local maxima) are given in Montreal Neurological Institute standard space. Local maxima shown were significant (p < 0.05) after family-wise error (FWE) correction for multiple voxel-wise comparisons within the pre-specified anatomical regions of interest (see text and Supplementary Figure S2).

**
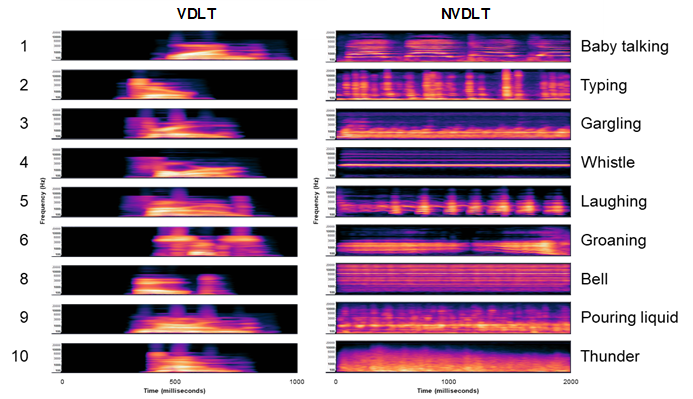
**

**Figure S1.** Spectrograms of the individual digits (left) and sounds (right) used in the verbal and non-verbal dichotic listening tests respectively.


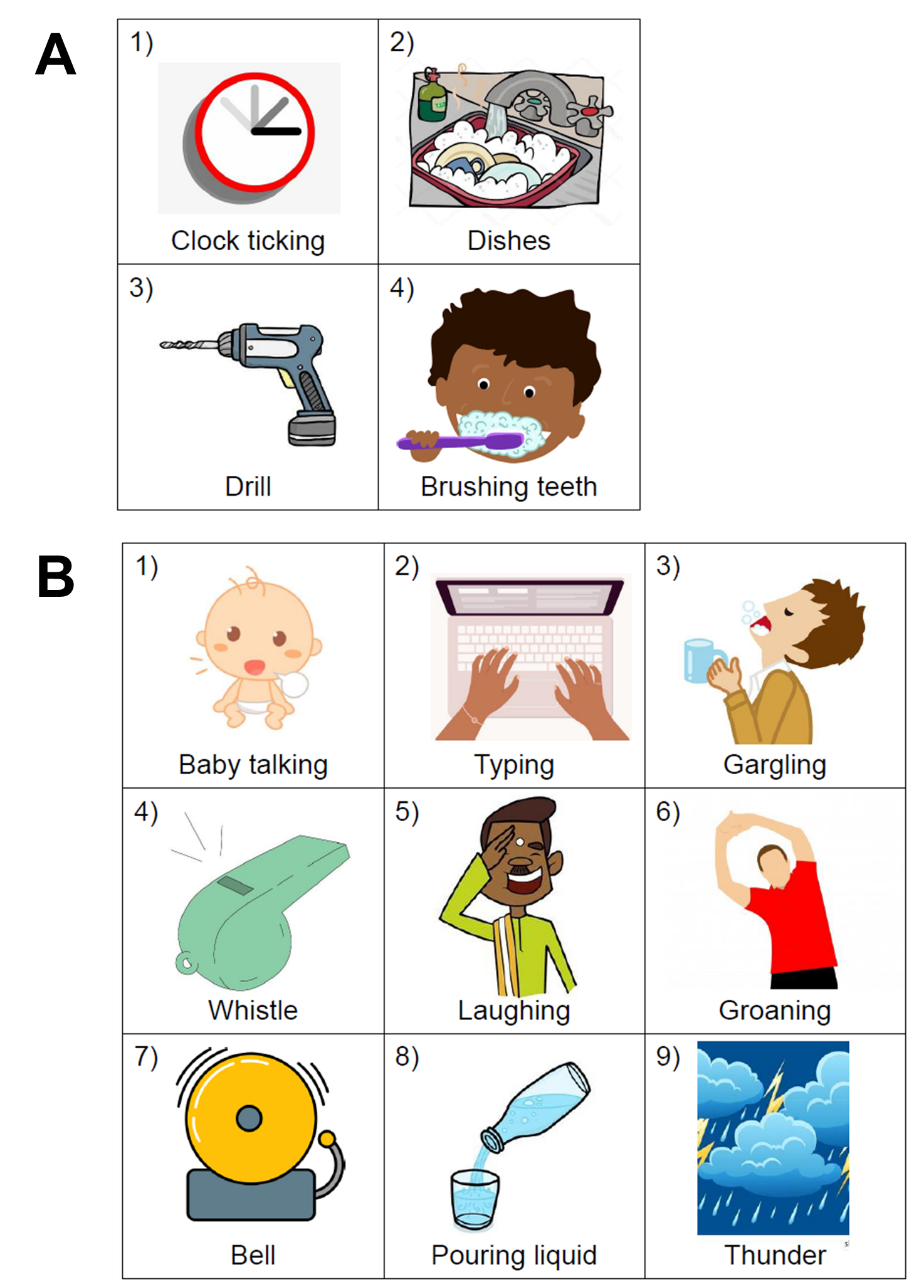


**Figure S2.** Cue cards used in the experiment for the non-verbal dichotic listening test. **A**) Cue card showing visual and written depictions of the sounds used in the practice trials. **B**) Cue card showing visual and written depictions of the sounds used in the main experimental trials. Participants had the relevant cue card in front of them for the duration of the task and could use this to give their response after each trial, but were instructed not to touch or point to any element of the card whilst the sounds were playing.

**
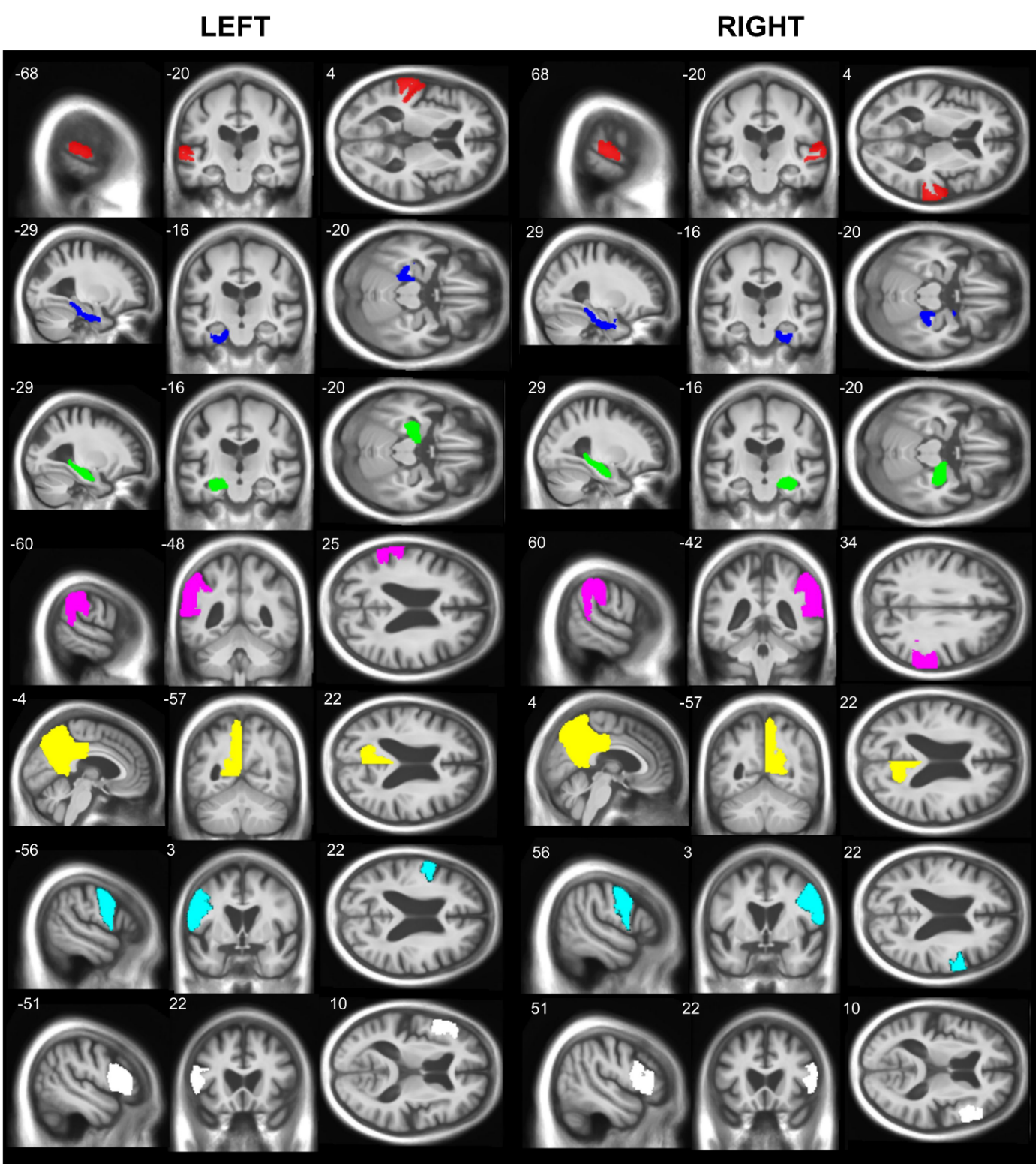
**

**Figure S3. Representative sections of neuroanatomical regions in both left and right cerebral hemisphere that were used for multiple voxel-wise comparisons correction in region-of-interest analyses** (see text). Regions are rendered on sagittal (left), coronal (middle) and axial (right) sections of the mean normalised brain template for the patient cohort; MNI coordinates of the plane of each section are shown. The neuroanatomical regions comprise (i) posterior superior temporal gyrus and planum temporale; (ii) parahippocampal gyrus; (iii) hippocampal gyrus; (iv) supramarginal gyrus; (v) posteromedial cortex; (vi) premotor cortex; and (vii) inferior frontal gyrus. Due to the asymmetry of these disease groups, regions in the left and right cerebral hemispheres were analysed separately. Anatomical volumes were derived from Oxford-Harvard cortical maps.

**
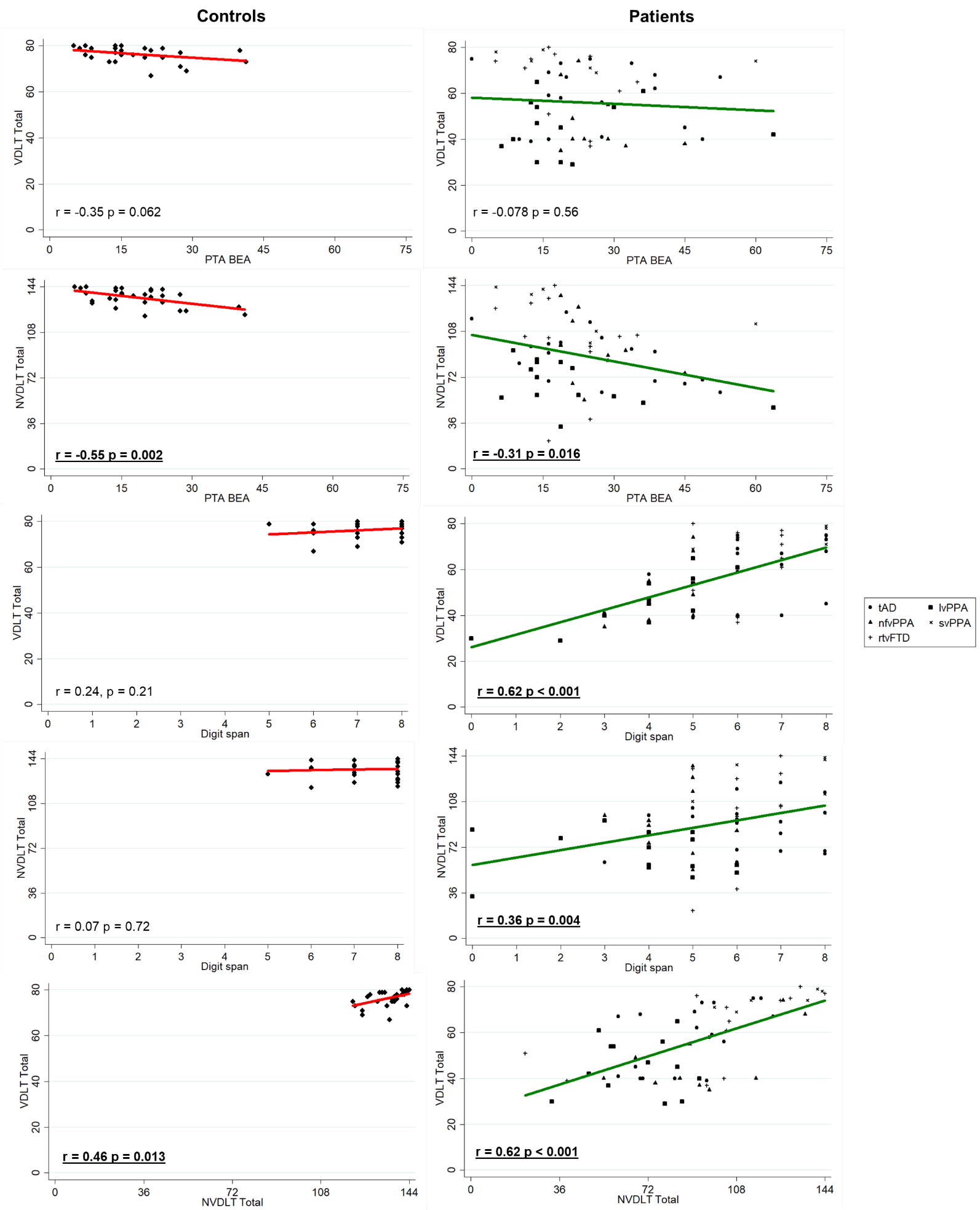
**

**Figure S4.** Scatter plots showing correlations within the healthy control (left panels) and combined patient groups (right panels). Diagnostic group membership is described in the key. Significant correlations are indicated in **bold underline**. tAD, typical Alzheimer’s disease; lvPPA, logopenic variant primary progressive aphasia; nfvPPA, nonfluent/agrammatic variant primary progressive aphasia; NVDLT, non-verbal dichotic listening test; PTA-BEA, pure-tone audiometry better ear average; svPPA, semantic variant primary progressive aphasia; VDLT, verbal dichotic listening test.
